# Deterministic stability and random behavior of a Hepatitis C model

**DOI:** 10.1371/journal.pone.0181571

**Published:** 2017-07-25

**Authors:** Mehmet Merdan, Zafer Bekiryazici, Tulay Kesemen, Tahir Khaniyev

**Affiliations:** 1 Department of Mathematical Engineering, Gumushane University, Gumushane, Turkey; 2 Department of Mathematics, Recep Tayyip Erdogan University, Rize, Turkey; 3 Department of Mathematics, Karadeniz Technical University, Trabzon, Turkey; 4 Department of Industrial Engineering, TOBB University of Economics and Technology, Ankara, Turkey; Shanxi University, CHINA

## Abstract

The deterministic stability of a model of Hepatitis C which includes a term defining the effect of immune system is studied on both local and global scales. Random effect is added to the model to investigate the random behavior of the model. The numerical characteristics such as the expectation, variance and confidence interval are calculated for random effects with two different distributions from the results of numerical simulations. In addition, the compliance of the random behavior of the model and the deterministic stability results is examined.

## Introduction

Hepatitis C is an infectious liver disease. The virus which causes this disease was identified in 1989 but the worldwide presence of the virus shows that it has been active for a much longer period. It is estimated that around 150 million people are chronically infected by the World Health Organization (WHO) reports [1]. Hepatitis C Virus (HCV) causes 3-4 million new infections per year. HCV infections occur in two basic stages, acute and chronic infections. The terms ‘acute’ and ‘chronic’ refer to the duration of the disease and not the severity. The illness can range from a mild illness which lasts less than a month to serious infections which can last several months, and even a lifetime in some cases [1]. The acute stage of the disease is largely asymptomatic and about one fifth of these cases resolve spontaneously due to adequate response to the HCV by the immune system. Less than one fifth of acute infections show mild symptoms like fatigue and jaundice. Infections that last up to 6 months are called ‘acute’ and acute infections have 1% mortality rate [2, 3]. Those that last longer are called ‘chronic’. Nearly 80% of HCV infections develop into the chronic stage which can last asymptomatic for more than 20 years [2, 4], [1]. The long period of asymptomatic infections makes the diagnosis of the disease difficult therefore hepatitis C is sometimes called the ‘silent epidemic’ [2, 3]. In about 30 years, more than one fifth of the infected develop cirrhosis and 1%-3% develop lung cancer. More than 300,000 people die yearly from diseases that are related with HCV [1, 2].

Stability analysis of the equilibrium points of the system provides a better understanding of the behavior of the system in a long period of time without the need to find the solutions of the model. Local stability of an equilibrium point suggests that if the system is close to this point, it will eventually reach the equilibrium state at this point. The global stability of an equilibrium point suggests that the system will reach equilibrium state at this point, whether it is close to this point or not [5]. Considering models in medicine, biology and etc., global stability of equilibrium points can indicate extinction or persistence of the disease according to the equilibrium point under consideration [6]. Local stability analysis examines the effects of small variations in each of the variables on the results of the model. Hence, a motivation for the random analysis of the model containing random effects in the parameters is to visualize the random behavior of the model, which can be linked with the stability of the equilibrium points under small changes in the conditions of the system.

The behavior of the solution of Hepatitis C virus model is examined by Ahmed and El-Saka [7]. The model in [7] is a fractional model which compares the results of the system for various powers of differentiation. Fractional calculus, with the use of its memory effect property, may provide useful information on the results of the model. However, we concentrate on the system of ordinary differential equations (*α* = 1), since we want to add random effects to the parameters and investigate the randomness of the event. Similar dynamical modeling studies can also be reviewed for an investigation of the framework of disease transmission models consisting of ordinary differential equations [8–12]. It should also be noted that the use of spatial effects may also provide useful results for analyzing Hepatitis C transmission dynamics [13–16]. These models use mathematical tools to guide and enhance studies in medicine, biology and etc. and with the addition of random effects, we intend to extend these analyses with the addition of a statistical point of view.

**Fig 1:**
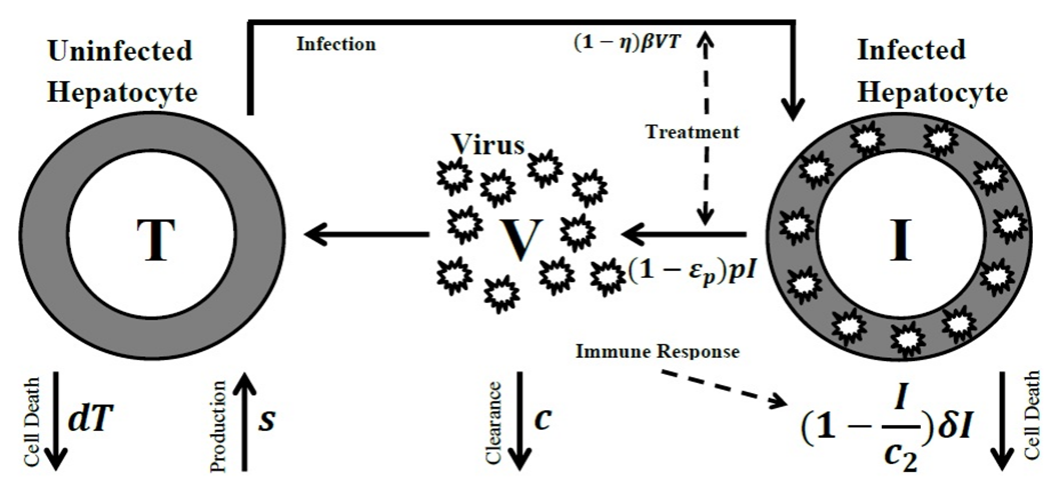
Flowchart of Model (1).

The components of the basic three-component model are uninfected hepatocytes, infected hepatocytes and the virus, which are denoted by *T*(*t*), *I*(*t*) and *V*(*t*), respectively. The flowchart of this model can be visualized as Fig 1, which has been obtained by a modification of the flowchart in [17].

$$\begin{array}{ccc} \frac{dT}{dt} & = & s-dT-(1-\eta )\beta VT,\\ \frac{dI}{dt} & = & \left(1-\eta \right)\beta VT-\delta I\left(1-\frac{I}{c_{2}}\right),\\ \frac{dV}{dt} & = & (1-\epsilon_{p})pI-cV. \end{array}$$ (1)

The parameters of the model describe the rates of change in the uninfected hepatocytes, infected hepatocytes and virus during treatment. *s* describes the constant production rate of uninfected hepatocytes per cell, while *d* and *β* describe their constant death and infection rates per cell. *δ* is the constant rate of death per cell for infected hepatocytes. *p* is the constant rate of production for viral particles per infected hepatocyte and *c* is their constant clearance rate per virion. The treatment effects are included in the model with two parameters *ϵ*_*p*_ and *η*, which describe the virion production blockage and new infection reduction respectively. For example, *ϵ*_*p*_ = 0.95 indicates 95% efficacy in blocking of virion production. *c*_2_ describes the rate of immunity response.

These parameters must be assigned values for the simulations of the Model (1). Values and descriptions of the parameters of model, which were obtained from [7] are explained in Table 1. Simulations are done with these parameter values above and the initial conditions *T*(0) = 2.4 × 10^6^, *I*(0) = 2.0 × 10^6^ and *V*(0) = 4.0 × 10^5^. Study motivation of this work is covered by the earlier study of Merdan and Khaniyev [18].

**Table 1:** Values and descriptions of the parameters of the model [7].

Parameter	Value	Description
*c*	6	Virion clearance rate
*d*	0.0026	Uninfected hepatocyte death rate
*β*	2.25 × 10^−7^	Uninfected hepatocyte infection rate
*δ*	0.26	Infected hepatocyte death rate
*s*	26000	Uninfected hepatocyte production rate
*ϵ*_*p*_	0.99	Efficacy of treatment (Blocking virion production)
*η*	0.95	Efficacy of treatment (Reducing new infections)
*p*	2.9	Virion production rate
*c*_2_	5 × 10^6^	Rate of immunity response

## Basic properties of the model

Firstly, the basic reproduction number and some properties of the model are examined. The disease-free equilibrium (DFE) for the System (1) is $E^{0}=\left(\frac{s}{d},0,0\right)$. The basic reproduction number is used for the analysis of the spread and control of the disease mathematically. It indicates whether the disease will spread through the community or be taken under control, which is very useful information. We will use the formula from [17, 19–22] to calculate this number for System (1). A similar study [23] can also be reffered to for the calculation of the equilibrium points of a similar model for HCV transmission. Let *X* = [*I*, *V*, *T*]^*T*^, then for System (1)

$$\begin{array}{ccc} & & \frac{dX}{dt}=F\left(X\right)-W\left(X\right),\\ & & F\left(X\right)=\left[\begin{array}{c} \left(1-\eta \right)\beta VT+\frac{\delta I^{2}}{c_{2}}\\ 0\\ 0 \end{array}\right],W\left(X\right)=\left[\begin{array}{c} \delta I\\ -(1-\epsilon_{p})pI+cV\\ -s+dT+(1-\eta )\beta VT \end{array}\right]. \end{array}$$

Jacobian of F at DFE and Jacobian of W are obtained respectively as follows: A straightforward calculation yields

$$\begin{array}{c} F\left(X\right)=\left[\begin{array}{cc} 0 & \frac{(1-\eta )\beta s}{d}\\ 0 & 0 \end{array}\right],V\left(X\right)=\left[\begin{array}{cc} \delta & 0\\ -(1-\epsilon_{p})p & c \end{array}\right]. \end{array}$$

The inverse of *V* is used with *F*(*X*) to obtain;

$$\begin{array}{c} V^{-1}\left(X\right)=\frac{1}{\delta c}\left[\begin{array}{cc} c & 0\\ (1-\epsilon_{p})p & \delta \end{array}\right]\Rightarrow FV^{-1}=\left[\begin{array}{cc} \frac{\left(1-\eta \right)\left(1-\epsilon_{p}\right)ps\beta }{\delta cd} & \frac{(1-\eta )\beta s}{cd}\\ 0 & 0 \end{array}\right]. \end{array}$$

The spectral Radius *R*_0_ is $R_{0}=\rho \left[FV^{-1}\right]=\frac{\left(1-\eta \right)\left(1-\epsilon_{p}\right)ps\beta }{\delta cd}$, as found in [20]. [24] can also be referred to for the calculation of the basc reproduction number.

**Proposition 1.** If *R*_0_ > 1, then there exists positive equilibria for the System (1) and one of these is the endemic equilibrium $E^{1}=\left(T_{1}^{*},I_{1}^{*},V_{1}^{*}\right)$, where $T_{1}^{*},I_{1}^{*},V_{1}^{*}$ can be defined as $T_{1}^{*}=\frac{c}{A}\left[\frac{\delta }{B}\left(1-\frac{I_{1}^{*}}{c_{2}}\right)\right]$, $I_{1}^{*}=\frac{-D+\sqrt{D^{2}-4E}}{2}$, $V_{1}^{*}=\frac{BI_{1}^{*}}{c}$ whereas *A* = (1 − *η*)*β*, *B* = (1 − *ϵ*_*p*_)*p*, $D=\frac{c_{2}}{\delta }\left(\frac{s}{R_{0}c_{2}}-\delta \right)$, $E=\left(1-\frac{1}{R_{0}}\right)\frac{sc_{2}}{\delta }$.

**Proof.** Let all the equations of System (1) equal to zero, then

$$\begin{array}{ccc} & & s-dT-(1-\eta )\beta VT=0,\\ & & \left(1-\eta \right)\beta VT-\delta I\left(1-\frac{I}{c_{2}}\right)=0,\\ & & (1-\epsilon_{p})pI-cV=0. \end{array}$$ (2)

From the last equation in Eq (2), we see that $V=\frac{(1-\epsilon_{p})pI}{c}$, when *c* ≠ 0. Replacing *V* in the second equation of Eq (2), we find $\delta I\left(1-\frac{I}{c_{2}}\right)=\left(1-\eta \right)\beta \frac{(1-\epsilon_{p})pI}{c}T$. Hence *T* is obtained from this expression as

$$\begin{array}{c} T=\frac{\delta c(1-\frac{I}{c_{2}})}{\left(1-\eta \right)\left(1-\epsilon_{p}\right)p\beta }. \end{array}$$ (3)

Substituting Eq (3) into the first equation of Eq (2), we get

$$\begin{array}{ccc} f(I) & = & s-d\frac{\delta c(1-\frac{I}{c_{2}})}{\left(1-\eta \right)\left(1-\epsilon_{p}\right)p\beta }-\left(1-\eta \right)\beta \frac{(1-\epsilon_{p})pI}{c}\frac{\delta c(1-\frac{I}{c_{2}})}{\left(1-\eta \right)\left(1-\epsilon_{p}\right)p\beta }\\ & = & s-\frac{\delta cd(1-\frac{I}{c_{2}})}{\left(1-\eta \right)\left(1-\epsilon_{p}\right)p\beta }-\delta I\left(1-\frac{I}{c_{2}}\right)=0. \end{array}$$

*f*(*I*) is a quadratic function, and notice that

$$\begin{array}{ccc} & & f\left(\frac{s}{d}\right)=s-\left(1-\frac{s}{dc_{2}}\right)\frac{s}{R_{0}}-\delta \frac{s}{d}\left(1-\frac{s}{dc_{2}}\right)>0\\ & & f\left(0\right)=s-\frac{\delta cd}{\left(1-\eta \right)\left(1-\epsilon_{p}\right)p\beta }=s-\frac{s}{R_{0}}=s\left(\frac{R_{0}-1}{R_{0}}\right)>0 \end{array}$$

where *R*_0_ > 1. Thus, *f*(*I*) = 0 has positive form and two roots. Therefore, the endemic equilibriums of the System (1) are given by

$$\begin{array}{c} E^{1,2}\left(T_{1,2}^{*},I_{1,2}^{*},V_{1,2}^{*}\right)=\left(\frac{c}{A}\left[\frac{\delta }{B}\left(1-\frac{I_{1,2}^{*}}{c_{2}}\right)\right],\frac{-D\pm \sqrt{D^{2}-4E}}{2},\frac{BI_{1,2}^{*}}{c}\right) \end{array}$$

with positive equilibrium *E*^1^ and *A* = (1 − *η*)*β*, *B* = (1 − *ϵ*_*p*_)*p*, $D=\frac{c_{2}}{\delta }\left(\frac{s}{R_{0}c_{2}}-\delta \right)$, $E=\left(1-\frac{1}{R_{0}}\right)\frac{sc_{2}}{\delta }$. Since $f\left(0\right)=s\left(\frac{R_{0}-1}{R_{0}}\right)<0$ when *R*_0_ < 1 and *s* > 0, the equation *f*(*I*) = 0 does not have any positive roots. However it can be seen that $I_{2}^{*}$ becomes negative for every value of *R*_0_, thus the equilibrium point *E*^2^ is biologically irrelevant and should be ignored.

## Local stability

In this part of the study, the local stability of the model is investigated for the equilibrium points.

**Theorem 1.** For the disease-free equilibrium point *E*^0^ of the System (1), we have the following:

1. *E*^0^ is locally asymptotically stable if *R*_0_ ≤ 1.
2. *E*^0^ is unstable if *R*_0_ > 1.

**Proof.** When evaluated at the point *E*^0^, System (1) has the Jacobian matrix

$$\begin{array}{c} J\left(E^{0}\right)=J_{0}=\left[\begin{array}{ccc} -d & 0 & -\frac{(1-\eta )\beta s}{d}\\ 0 & -\delta & \frac{(1-\eta )\beta s}{d}\\ 0 & (1-\epsilon_{p})p & -c \end{array}\right]. \end{array}$$

where $\frac{(1-\eta )\beta s}{d}=F$, (1 − *ϵ*_*p*_)*p* = *G*. The characteristic equation of *J*_0_ is given by *λ*^3^ + *Q*_1_*λ*^2^ + *Q*_2_*λ* + *Q*_3_ = 0, where

$$\begin{array}{ccc} Q_{1} & = & c+d+\delta >0,\\ Q_{2} & = & c\left(\delta +d\right)+\delta d-GF=c\left(\delta +d\right)+\delta d-\delta cR_{0}>0,\\ Q_{3} & = & d\left(c\delta -GF\right)=d\left(c\delta -\delta cR_{0}\right)>0. \end{array}$$

The Routh-Hurwitz criterion for the cubic equation is as follows:

$$\begin{array}{ccc} Q_{1}Q_{2}-Q_{3} & = & \left(c+d+\delta )[c(\delta +d)+\delta d-GF]-d(c\delta -GF\right)\\ & = & (d+\delta )[c(c+d+\delta )+\delta d]-GF(c+\delta )>0. \end{array}$$

Thus, the Routh-Hurwitz criterion is satisfied. So, the System (1) is locally asymptotically stable in the neighborhood of the disease-free equilibrium (DFE) *E*^0^. If *R*_0_ > 1, then *Q*_2_ < 0, implying that *E*^0^ is unstable. The referred study [20] can be reviewed for further notes on the stability of the disease-free equilibrium.

**Theorem 2.** The endemic equilibrium point $E^{1}\left(T_{1}^{*},I_{1}^{*},V_{1}^{*}\right)$ of the System (1) is locally asymptotically stable when *R*_0_ > 1 and unstable otherwise.

**Proof.** When evaluated at the point *E*^1^, System (1) has the Jacobian matrix

$$\begin{array}{c} J\left(E^{1}\right)=J_{1}=\left[\begin{array}{ccc} -d-\left(1-\eta \right)\beta V_{1}^{*} & 0 & -\left(1-\eta \right)\beta T_{1}^{*}\\ \left(1-\eta \right)\beta V_{1}^{*} & -\delta +\frac{2\delta I_{1}^{*}}{c_{2}} & \left(1-\eta \right)\beta T_{1}^{*}\\ 0 & (1-\epsilon_{p})p & -c \end{array}\right]. \end{array}$$

where (1 − *η*)*β* = *A*, (1 − *ϵ*_*p*_)*p* = *B*. The characteristic equation of *J*_1_ is given by $\lambda^{3}+Q_{1}^{*}\lambda^{2}+Q_{2}^{*}\lambda +Q_{3}^{*}=0$, where

$$\begin{array}{ccc} Q_{1}^{*} & = & c+\delta +d+AV_{1}^{*}-\frac{2\delta I_{1}^{*}}{c_{2}},\\ Q_{2}^{*} & = & c\delta -\frac{2\delta cI_{1}^{*}}{c_{2}}-\frac{\delta cdR_{0}T_{1}^{*}}{s}+\left(d+AV_{1}^{*}\right)\left(c+\delta -\frac{2\delta I_{1}^{*}}{c_{2}}\right)\\ Q_{3}^{*} & = & c\left(d+AV_{1}^{*}\right)\left(\delta -\frac{2\delta I_{1}^{*}}{c_{2}}\right)-ABdT_{1}^{*}=c\delta \left(d+AV_{1}^{*}\right)\left(1-\frac{2I_{1}^{*}}{c_{2}}\right)-\frac{\delta cd^{2}R_{0}T_{1}^{*}}{s}. \end{array}$$

The Routh-Hurwitz criterion for the cubic equation is as follows:

$$\begin{array}{ccc} Q_{1}^{*}Q_{2}^{*}-Q_{3}^{*} & = & \left(c+\delta +d+AV_{1}^{*}-\frac{2\delta I_{1}^{*}}{c_{2}}\right)\\ & & \left[c\delta -\frac{2\delta cI_{1}^{*}}{c_{2}}-\frac{\delta cdR_{0}T_{1}^{*}}{s}+\left(d+AV_{1}^{*}\right)\left(c+\delta -\frac{2\delta I_{1}^{*}}{c_{2}}\right)\right]\\ & & -c\delta \left(d+AV_{1}^{*}\right)\left(1-\frac{2I_{1}^{*}}{c_{2}}\right)-\frac{\delta cd^{2}R_{0}T_{1}^{*}}{s}>0. \end{array}$$

When *R*_0_ < 1, it is easy to see that $Q_{1}^{*}>0$, $Q_{2}^{*}<0$, $Q_{3}^{*}<0$ and $Q_{1}^{*}Q_{2}^{*}-Q_{3}^{*}>0$. Thus, by Routh-Hurwitz criterion, the endemic equilibrium point $E^{1}\left(T_{1}^{*},I_{1}^{*},V_{1}^{*}\right)$ is unstable.

## Global stability

The global stabilities of the disease-free equilibrium *E*^0^ and the endemic equilibrium *E*^1^ of the Hepatitis C Virus transmission model are investigated in this section [25–27].

**Theorem 3.** The disease-free equilibrium *E*^0^ is globally asymptotically stable in the positively variant set Ω for *R*_0_ < 1.

**Proof.** Consider the following Lyapunov function *U*(*t*) = (1 − *ϵ*_*p*_)*pI* + *δV* [28]. Calculating the derivative $\frac{dU(t)}{dt}$ for the solutions of System (1), we get

$$\begin{array}{ccc} \frac{dU(t)}{dt} & = & \left(1-\epsilon_{p}\right)p\left(\frac{dI}{dt}\right)+\delta \left(\frac{dV}{dt}\right)\\ & = & \left(1-\epsilon_{p}\right)p\left(\left(1-\eta \right)\beta VT-\delta I\left(1-\frac{I}{c_{2}}\right)\right)+\delta \left(\left(1-\epsilon_{p}\right)pI-cV\right)\\ & = & \left(1-\eta \right)\left(1-\epsilon_{p}\right)p\beta VT-\delta cV+\frac{\delta }{c_{2}}I^{2}\leq V\left[\left(1-\eta \right)\left(1-\epsilon_{p}\right)p\beta \left(\frac{s}{d}\right)-\delta c\right]\\ & = & \frac{V}{d}\left[R_{0}\delta cd-\delta cd\right]=V\delta c\left(R_{0}-1\right). \end{array}$$

Provided that *R*_0_ < 1, we find that $\frac{dU(t)}{dt}\leq 0$, which, considering LaSalle’s invariance principle [29], indicates that the disease-free equilibrium *E*^0^ is globally asymptotically stable.

In the following, we deal with the geometric approach developed in [27] for the proof of the global stability of endemic equilibrium point *E*^1^. A simple sufficient condition guarantees the global asymptotical stability of the epidemic equilibrium *E*^1^. We begin with a summary of the geometric approach.

Consider the autonomous dynamical system:

$$\begin{array}{c} \frac{dx}{dt}=f\left(x\right), \end{array}$$ (4)

where *f*: *D* → *R*^*n*^, *D* ⊂ *R*^*n*^ is an open set and *f* ∈ *C*^1^(*D*).

Assume that the following hypothesis hold [25]:

- (*H*_1_): Ω is simply connected;
- (*H*_2_): A compact absorbing set *K* ⊂ Ω exists;
- (*H*_3_): A unique equilibrium *x*_∗_ exists for the differential Eq (4) in Ω,

where Ω is the region where the model makes biological sense.

**Lemma 1.** ([30]) Assume that (*H*_2_) and (*H*_3_) are satisfied and that System (4) satisfies a Bendixson criterion which is robust under *C*^1^ local perturbations of *f*(*x*) for all non-equilibrium non-wandering points of System (4). If *x*_∗_ is stable then it is globally stable in *D*.

Let $x\to p\left(x\right)\in \left(\begin{array}{c} n\\ 2 \end{array}\right)\times \left(\begin{array}{c} n\\ 2 \end{array}\right)$ be a matrix-valued function which is *C*^1^ for *x* ∈ *D*. Assume that *p*^−1^(*x*) exists and is continuous for *x* ∈ *K*. Define q as follows $q=lim_{t\to \infty }supsup_{x_{0}\in K}\frac{1}{t}\int_{0}^{t}\mu \left(B\left(x\left(s,x_{o}\right)\right)\right)ds$, where *B* = *p*_*f*_*p*^−1^ + *pJ*^[2]^*p*^−1^. Thus, *p*_*f*_ is the matrix given by,

$$\begin{array}{c} {\left(p_{ij}\left(x\right)\right)}_{f}={\left(\frac{\partial p_{ij}\left(x\right)}{\partial x}\right)}^{T}\cdot f\left(x\right)=▿p_{ij}\left(x\right)\cdot f\left(x\right) \end{array}$$

and *J*^[2]^ is the second additive compound matrix of the Jacobian matrix (*J*(*x*) = *Df*(*x*)), while *μ*(*B*) is the Lozinski measure of *B* with respect to a vector norm |.| in $R^{n},N=C_{n}^{2}$ and is defined by

$$\begin{array}{c} \mu \left(B\right)=lim_{h\to 0^{+}}\frac{|I+hB|-1}{h}. \end{array}$$

The following result for global stability is proved by [27].

**Lemma 2.** ([30]) Suppose that *D* is simply connected and that (*H*_2_) and (*H*_3_) are satisfied. Then the unique equilibrium *x*_∗_ of System (4) is globally stable in *D* if *R*_0_ < 0.

The following result can be found with the lemmas above.

**Theorem 4.** The endemic equilibrium $E^{1}=\left(T_{1}^{*},I_{1}^{*},V_{1}^{*}\right)$ of System (1) is globally asymptotically stable in Ω if *R*_0_ > 1.

**Proof.** Firstly, we consider the Jacobian matrix of System (1)

$$\begin{array}{c} J=[\begin{array}{ccc} -d-(1-\eta )\beta V & 0 & -(1-\eta )\beta T\\ (1-\eta )\beta V & -\delta +\frac{2\delta I}{c_{2}} & (1-\eta )\beta T\\ 0 & (1-\epsilon_{p})p & -c \end{array}]. \end{array}$$

Its second additive compound matrix is

$$\begin{array}{c} J^{\left[2\right]}=\left[\begin{array}{ccc} -d-\delta +\frac{2\delta I}{c_{2}}-\left(1-\eta \right)\beta V & (1-\eta )\beta T & (1-\eta )\beta T\\ (1-\epsilon_{p})p & -c-d-(1-\eta )\beta V & 0\\ 0 & (1-\eta )\beta V & -c-\delta +\frac{2\delta I}{c_{2}} \end{array}\right]. \end{array}$$

Let $p\left(x\right)=P\left(T,I,V\right)=diag\left(1,\frac{I}{V},\frac{I}{V}\right)$, then

$$\begin{array}{ccc} & & p_{f}=diag\left(0,\frac{\dot{I}V-I\dot{V}}{V^{2}},\frac{\dot{I}V-I\dot{V}}{V^{2}}\right),\;p_{f}p^{-1}=diag\left(0,\frac{\dot{I}}{I}-\frac{\dot{V}}{V},\frac{\dot{I}}{I}-\frac{\dot{V}}{V}\right),\\ & & pJ^{\left[2\right]}p^{-1}=\left[\begin{array}{ccc} -d-\delta +\frac{2\delta I}{c_{2}}-\left(1-\eta \right)\beta V & \frac{\left(1-\eta \right)\beta V^{2}T}{I} & \frac{(1-\eta )\beta TV}{I}\\ \frac{(1-\epsilon_{p})pI}{V} & -c-d-(1-\eta )\beta V & 0\\ 0 & (1-\eta )\beta V & -c-\delta +\frac{2\delta I}{c_{2}} \end{array}\right]. \end{array}$$

The matrix $B=p_{f}p^{-1}+pJ^{\left[2\right]}p^{-1}=\left[\begin{array}{cc} B_{11} & B_{12}\\ B_{21} & B_{22} \end{array}\right]$, where

$$\begin{array}{ccc} & & B_{11}=-d-\delta +\frac{2\delta I}{c_{2}}-\left(1-\eta \right)\beta V,\;B_{12}=\left(\frac{\left(1-\eta \right)\beta V^{2}T}{I},\frac{(1-\eta )\beta TV}{I}\right)\\ & & B_{21}={\left(\frac{(1-\epsilon_{p})pI}{V},0\right)}^{T},\\ & & B_{22}=\left[\begin{array}{cc} -c-d-\left(1-\eta \right)\beta V+\frac{\dot{I}}{I}-\frac{\dot{V}}{V} & 0\\ (1-\eta )\beta V & -c-\delta +\frac{2\delta I}{c_{2}}+\frac{\dot{I}}{I}-\frac{\dot{V}}{V} \end{array}\right]. \end{array}$$

Let (*a*_1_, *a*_2_, *a*_3_) be a vector in $R^{3}$. Its norm *L*^1^ ∥ . ∥ is defined as

$$\begin{array}{c} ∥\left(a_{1},a_{2},a_{3}\right)∥=max\{|a_{1}\left|,\right|a_{2}\left|+\right|a_{3}\left|\right\}. \end{array}$$

Denote the Lozinski measure with respect to this norm by *μ*(*B*). It follows from the notation in [31] that we have *μ*(*B*) ≤ *sup*{*g*_1_, *g*_2_}, where

$$\begin{array}{c} g_{1}=\mu_{1}\left(B_{11}\right)+|B_{12}|,\;g_{2}=\left|B_{21}\right|+\mu_{1}\left(B_{22}\right). \end{array}$$

|*B*_12_|, |*B*_21_| are matrix norms with respect to the *L*^1^ vector norm, and *μ*_1_ denotes the Lozinski measure with respect to the *L*^1^ norm. Then

$$\begin{array}{ccc} & & \mu_{1}\left(B_{11}\right)=-d-\delta +\frac{2\delta I}{c_{2}}-\left(1-\eta \right)\beta V,\;|B_{12}|=\frac{(1-\eta )\beta TV}{I},\;\left|B_{21}\right|=\frac{(1-\epsilon_{p})pI}{V},\\ & & \mu_{1}\left(B_{22}\right)=max\left\{-c-d-\left(1-\eta \right)\beta V+\frac{\dot{I}}{I}-\frac{\dot{V}}{V},-c-\delta +\frac{2\delta I}{c_{2}}+\frac{\dot{I}}{I}-\frac{\dot{V}}{V}\right\}\\ & & \leq \frac{\dot{I}}{I}-\frac{\dot{V}}{V}-c-h, \end{array}$$

where $h=min\{d+\left(1-\eta \right)\beta V,\delta -\frac{2\delta I}{c_{2}}\}>0$. Therefore, we have

$$\begin{array}{ccc} & & g_{1}=-d-\delta +\frac{2\delta I}{c_{2}}-\left(1-\eta \right)\beta V+\frac{(1-\eta )\beta TV}{I},\\ & & g_{2}\leq \frac{(1-\epsilon_{p})pI}{V}+\frac{\dot{I}}{I}-\frac{\dot{V}}{V}-c-h. \end{array}$$

From the equation System (1), we get

$$\begin{array}{c} \frac{\dot{I}}{I}=\frac{(1-\eta )\beta TV}{I}-\delta +\frac{\delta I}{c_{2}},\;\frac{\dot{V}}{V}=\frac{(1-\epsilon_{p})pI}{V}-c. \end{array}$$

Then we have

$$\begin{array}{c} g_{1}=\frac{\dot{I}}{I}-d+\frac{\delta I}{c_{2}}-\left(1-\eta \right)\beta V\leq \frac{\dot{I}}{I}-d,\;g_{2}\leq \frac{\dot{I}}{I}-h. \end{array}$$

Furthermore, we obtain $\mu \left(B\right)\leq sup\left\{g_{1},g_{2}\right\}\leq \frac{\dot{I}}{I}-d$. Along each solution (*T*(*t*), *I*(*t*), *V*(*t*)) of System (1) with (*T*(0), *I*(0), *V*(0)) ∈ *K*, where *K* is the compact absorbing set, we have

$$\begin{array}{c} \frac{1}{t}\int_{0}^{t}\mu \left(B\right)ds\leq \frac{1}{t}\int_{0}^{t}\left(\frac{\dot{I}}{I}-d\right)ds=\frac{1}{t}ln\frac{I(t)}{I(0)}-d, \end{array}$$

which implies

$$\begin{array}{ccc} q & = & lim_{t\to \infty }supsup_{x_{0}\in K}\frac{1}{t}\int_{0}^{t}\mu \left(B\left(x\left(s,x_{o}\right)\right)\right)ds\\ & \leq & lim_{t\to \infty }supsup_{x_{0}\in K}\frac{1}{t}ln\frac{I(t)}{I(0)}-d\leq -\frac{d}{2}<0. \end{array}$$

As a result, endemic equilibrium $E^{1}=\left(T_{1}^{*},I_{1}^{*},V_{1}^{*}\right)$ of System (1) is globally asymptotically stable in Ω if *R*_0_ > 1.

## Random behavior of the model

We investigate the behavior of the randomized components to visualize the effects of small variations on the model output. The parameters in the deterministic System (1) are added random effects to investigate the random characteristics of the model. A random analysis of models using random differential equations with random parameters has been used before for a model of avian-influenza by Merdan and Khaniyev in 2008 and for bacterial resistance by Merdan et al., which was the motivation for the method used in this work [18, 32]. The random model is analyzed to investigate the numerical characteristics of the event and thus comment on the random behavior of the model components. The randomness in the parameters of the model can be linked to the stability of the deterministic model since the stability of the equilibrium points are essentially the ability of the system of maintain its position on these points under small variations. We use normally and symmetrically distributed random effects to model real lifer variations in the parameters of the model. Normal distribution is commonly used for random variables with unknown distributions since the central limit theorem states that a sufficiently large number of independent random variables will be approximately normally distributed under certain conditions. A triangularly distributed random variable has a high probability around its mean and the probability decreases for values that are far away from the mean. A symmetrical triangular distribution and a normal distribution were used for the random effects since they are similar in the above mentioned sense and hence a comparison can be made for the randomness of the results.

### 0.1 Investigation of the model under normally varying random effects

The parameters *s*, *d*, *η*, *β*, *δ*, *c*_2_, *ϵ*_*p*_, *p* and *c* are considered to be random variables with normal distribution in order to investigate the model under normally distributed random effects.

Using the “randn” command in MATLAB, which generates random variables with standard normal distribution, we can generate random variables *s*, *d*, *η*, *β*, *δ*, *c*_2_, *ϵ*_*p*_, *p* and *c* which have normal distribution. These generated random variables will have the following forms: *c* = *c*_0_ + *σ*_1_*η*_1_, *d* = *d*_0_ + *σ*_2_*η*_2_, *β* = *β*_0_ + *σ*_3_*η*_3_, *δ* = *δ*_0_ + *σ*_4_*η*_4_, *s* = *s*_0_ + *σ*_5_*η*_5_, *ϵ*_*p*_ = *ϵ*_*p*_0__ + *σ*_6_*η*_6_, *η* = *η*_0_ + *σ*_7_*η*_7_, *p* = *p*_0_ + *σ*_8_*η*_8_, *c*_2_ = *c*_2_0__ + *σ*_9_*η*_9_, where the random variables $\eta_{i},i=\bar{1,9}$ are independent random variables with standard normal distribution and $\sigma_{i},i=\bar{1,9}$ are the corresponding deviations used for each of the parameters *s*, *d*, *η*, *β*, *δ*, *c*_2_, *ϵ*_*p*_, *p* and *c*. The following values will be used for the deviations of parameters:

$$\begin{array}{cc} & c=c_{0}+1.0\times 10^{-1}\times \eta_{1},\;d=d_{0}+1.0\times 10^{-4}\times \eta_{2},\;\beta =\beta_{0}+1.0\times 10^{-8}\times \eta_{3},\\ & \delta =\delta_{0}+1.0\times 10^{-2}\times \eta_{4},\;s=s_{0}+1.0\times 10^{+3}\times \eta_{5},\;\epsilon_{p}=\epsilon_{p_{0}}+1.0\times 10^{-2}\times \eta_{6},\\ & \eta =\eta_{0}+1.0\times 10^{-2}\times \eta_{7},\;p=p_{0}+1.0\times 10^{-1}\times \eta_{8},\;c_{2}=c_{2_{0}}+1.0\times 10^{+5}\times \eta_{9}. \end{array}$$

As it can be seen in the list above, normally distributed random variables, which are denoted as $\eta_{i},i=\bar{1,9}$, are added to the initial values of the parameters *s*, *d*, *η*, *β*, *δ*, *c*_2_, *ϵ*_*p*_, *p* and *c*, which are denoted by the zero-indexed parameter, therefore forming the new parameters which have normal distribution. The deviations of the random effects are determined to be powers of 10, so that the random effect added to the initial values of the parameters is around 1% to 4.4% of the initial value.

Replacing the parameters in the model with the new random variables listed above gives the system of random differential equations below:

$$\begin{array}{ccc} \frac{dT}{dt} & = & \left(s_{0}+1000\eta_{5}\right)-\left(d_{0}+0.0001\eta_{2}\right)T\\ & & +\left(1-\left(\eta_{0}+0.01\eta_{7}\right)\right)\left(\beta_{0}+0.00000001\eta_{3}\right)VT,\\ \frac{dI}{dt} & = & \left(1-\left(\eta_{0}+0.01\eta_{7}\right)\right)\left(\beta_{0}+0.00000001\eta_{3}\right)VT\\ & & -\left(\delta_{0}+0.01\eta_{4}\right)I\left(1-\frac{I}{\left(c_{2_{0}}+100000\times \eta_{9}\right)}\right),\\ \frac{dV}{dt} & = & \left(1-\left(\epsilon_{p_{0}}+0.01\eta_{6}\right)\right)\left(p_{0}+0.1\eta_{8}\right)I-\left(c_{0}+0.1\eta_{1}\right)V. \end{array}$$ (5)

MATLAB is used to obtain results for the model under random effects with 10^5^ simulations. The random system produces deterministic differential equations which are assigned different coefficients for every trial of the event.

Note that a random variable is a measureable function from the set of possible events to real numbers *R* meaning it is a real valued function. Hence for every trial of the event, the random variables produce different real numbers according to their random distribution. For the random model, this would mean that we would get a different set of differential equations for various trials which would all be deterministic differential equations with different valued coefficients. Since the random model produces deterministic equations with variations in the set of parameters, the random analysis of the behavior of model components will be based on the statistical properties of the numerical solutions of these deterministic equations. Using 10^5^ simulations of the numerical solutions of the equations, comments are made on the numerical characteristics of the model with random coefficients.

### 0.2 Simulation results for normal distribution

#### 0.2.1 Expectations

*T*(*t*), *I*(*t*) and *V*(*t*) under random effects are random variables. Hence, their moments can be evaluated using the law of large numbers.

$$\begin{array}{c} \hat{E}\left(T\left(t\right)\right)=\frac{1}{N}\sum_{i=1}^{N}T_{i}\left(t\right) \end{array}$$

**Fig 2:**
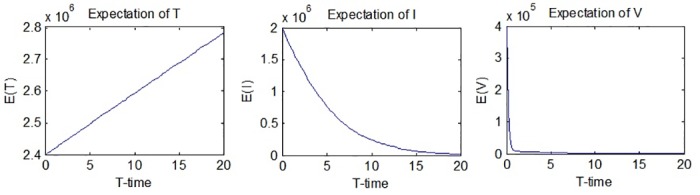
Expectations of *T*(*t*), *I*(*t*) and *V*(*t*).

*T*_*i*_(*t*), *i* = 1, …, *N* are the results obtained from the simulation of the process *T*(*t*). The graphs of *E*(*T*(*t*)), *E*(*I*(*t*)) and *E*(*V*(*t*)) are given in Fig 2.

Fig 2 suggests that the expectation of *T*(*t*) will go up while the expectations of *I*(*t*) and *V*(*t*) will go down. (2782200, 20) and (2400000, 0) are the max. and min. values for the expectation of *T*(*t*) respectively, meaning that the expected value increases from the beginning until the end. The expected values of the number of infected hepatocytes and virions both have decreasing behavior, although it can be seen that the decrease of the expected value of the number of virions is much more rapid. (2000000, 0) and (18783, 20) are the max. and min. values for the expectation of *I*(*t*), while (400000, 0) and (95.0005, 20) are the max. and min. values for the expectation of *V*(*t*).

#### 0.2.2 Variances

**Fig 3:**
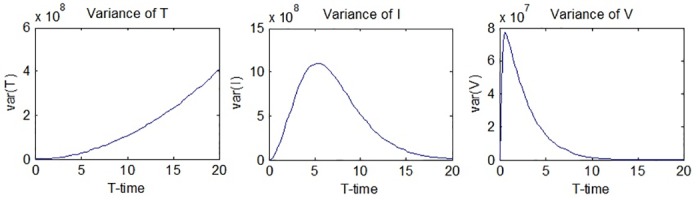
Variances of *T*(*t*), *I*(*t*) and *V*(*t*).

The graphs of *var*(*T*(*t*)), *var*(*I*(*t*)) and *var*(*V*(*t*)) are given in Fig 3.

It can be seen from Fig 3 that the variance of the number of uninfected hepatocytes, *var*(*T*(*t*)), increases throughout the process, while the rate of increase becomes slightly larger as the process continues. Fig 3 also suggests that The variances of the number of infected hepatocytes and virions show rapid increases in the beginning of the process but start to decrease after a while. The maximum and minimum values of the variances are obtained from the simulations in MATLAB as follows: *max*(*var*(*T*(*t*))) = 4.0603 × 10^8^ is obtained at *t* = 20, while *min*(*var*(*T*(*t*))) = 0 is obtained at *t* = 0. *max*(*var*(*I*(*t*))) = 1.1049 × 10^9^ at *t* = 5.2, while *min*(*var*(*I*(*t*))) = 0 is obtained at *t* = 0. *max*(*var*(*V*(*t*))) = 7.67 × 10^7^ is obtained at *t* = 0.6, while *min*(*var*(*V*(*t*))) = 0 is obtained at *t* = 20. Hence, it can be said that the randomness in the results for *I*(*t*) and *V*(*t*) are expected to reach a maximum level in the observed time interval and then fall down to zero again.

#### 0.2.3 Confidence intervals

**Fig 4:**
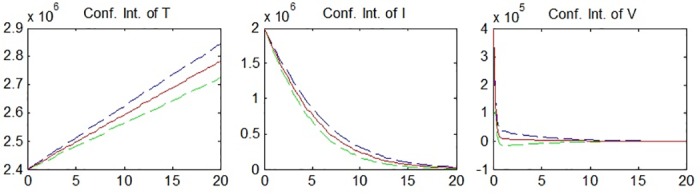
Confidence intervals of *T*(*t*), *I*(*t*) and *V*(*t*).

The confidence intervals of *T*(*t*), *I*(*t*) and *V*(*t*) are found in the form of [*E*(*T*(*t*)) − *Kσ*(*T*(*t*)), *E*(*T*(*t*)) + *Kσ*(*T*(*t*))] by using the expected values and standard deviations which were previously calculated. For *K* = 3, confidence intervals are given at approximately 99%, meaning that there is 99% probability that the given interval includes the real value of *T*(*t*). The confidence intervals can be seen in Fig 4 (The dashed lines are the upper and the lower limits of the intervals.).

Fig 4 shows that the confidence intervals, in accordance with the results for the variances, become wider in the process for *I*(*t*) and *V*(*t*), before narrowing down again. The extremum values of the confidence intervals are (2842600, 20) and (2400000, 0) for *T*(*t*), (2000000, 0) and (7431.9, 20) for *I*(*t*) and (400000, 0) and (0, 20) for *V*(*t*) (The lower limit of the confidence intervals are calculated by subtracting 3 standard deviations from the means, thus any negative value obtained must be ignored since it is biologically irrelevant).

### 0.3 Investigation of the model under triangularly varying random effects

The parameters are considered to be random variables with symmetrical triangular distribution in the interval (−1, 1) in order to investigate the model. Using the property above and the ‘rand’ command in MATLAB, which generates uniformly distributed random variables from (0, 1), we can generate random variables which have symmetrical triangular distribution in the interval (−1, 1). A similar modeling approach will produce the system under triangular effects as:

$$\begin{array}{ccc} \frac{dT}{dt} & = & s_{0}+0.1\left(\eta_{11}-\eta_{12}\right)-d_{0}+0.0001\left(\eta_{21}-\eta_{22}\right)T\\ & & +\left(1-\eta_{0}+0.01\left(\eta_{71}-\eta_{72}\right)\right)\left(\beta_{0}+0.00000001\left(\eta_{31}-\eta_{32}\right)\right)VT,\\ \frac{dI}{dt} & = & \left(1-\eta_{0}+0.01\left(\eta_{71}-\eta_{72}\right)\right)\left(\beta_{0}+0.00000001\left(\eta_{31}-\eta_{32}\right)\right)VT\\ & & -\left(\delta_{0}+0.01\left(\eta_{41}-\eta_{42}\right)\right)I\left(1-\frac{I}{c_{2_{0}}+100000\times \left(\eta_{91}-\eta_{92}\right)}\right),\\ \frac{dV}{dt} & = & \left(1-\epsilon_{p_{0}}+0.01\left(\eta_{61}-\eta_{62}\right)\right)\left(p_{0}+0.1\left(\eta_{81}-\eta_{82}\right)\right)I-\left(c_{0}+0.1\left(\eta_{11}-\eta_{12}\right)\right)V. \end{array}$$ (6)

Here, the random variables $\eta_{ij},i=\bar{1,9},j=1,2$ are uniformly distributed independent random variables from (0, 1), so that their difference can produce independent symmetrical triangular random variables in (−1, 1). Monte Carlo method is used in MATLAB to obtain results for the model under random effects. Simulations are repeated more than 10^5^ times in order to obtain accurate results.

### 0.4 Simulation results for triangular distribution

#### 0.4.1 Expectations

**Fig 5:**
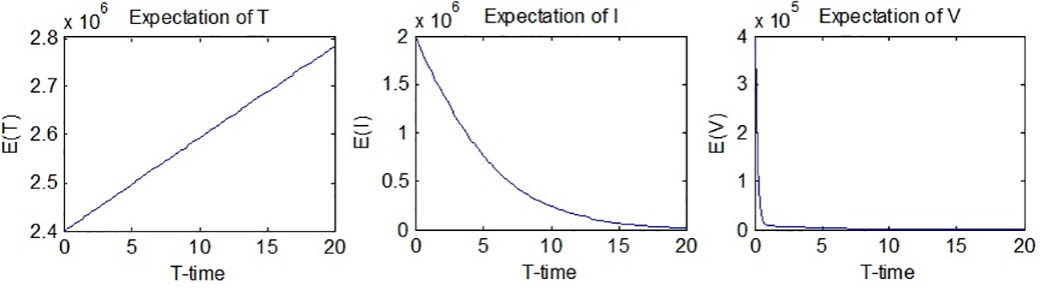
Expectations of *T*(*t*), *I*(*t*) and *V*(*t*).

Taking advantage of the law of large numbers, the expectations of *T*(*t*), *I*(*t*) and *V*(*t*) are evaluated similarly to the case of normally distributed random effects. The results for *E*(*T*(*t*)), *E*(*I*(*t*)) and *E*(*V*(*t*)) are given in Fig 5.

A similarity between the results of the expectations for the normally and triangularly distributed effects can be seen (Figs 2 and 5). The only difference between the results in these two cases are the extreme values of the expectations. Fig 5 shows that that the expectation of *T*(*t*) will go up while the expectations of *I*(*t*) and *V*(*t*) will go down through the observed time interval. (2782100, 20) and (2400000, 0) are the max. and min. values of *T*(*t*), respectively. (2000000, 0) and (18467, 20) are the max. and min. values of *I*(*t*), respectively. (400000, 0) and (93.387, 20) are the max. and min. values of *V*(*t*), respectively. The expectations under triangular effects match the solution curves of *T*(*t*), *I*(*t*) and *V*(*t*), which was also the case in normally distributed effects.

#### 0.4.2 Variances

**Fig 6:**
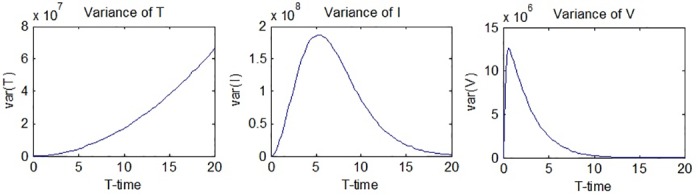
Variances of *T*(*t*), *I*(*t*) and *V*(*t*).

The graphs of *var*(*T*(*t*)), *var*(*I*(*t*)) and *var*(*V*(*t*)) are given in Fig 6.

While the shapes of the graphs for *var*(*T*(*t*)), *var*(*I*(*t*)) and *var*(*V*(*t*)) are the same for both normally and triangularly distributed effects (Figs 3 and 6), there is considerable difference in the values of these variances. It can be said that the variances show similar behavior, but with different values. The minimum and maximum values for the variances are as follows: *max*(*var*(*T*(*t*))) = 6.7858 × 10^7^ is obtained at *t* = 20, while *min*(*var*(*T*(*t*))) = 0 is obtained at *t* = 0. *max*(*var*(*I*(*t*))) = 1.8583 × 10^8^ is obtained at *t* = 5.4, while *min*(*var*(*I*(*t*))) = 0 is obtained at *t* = 0. *max*(*var*(*V*(*t*))) = 1.2817 × 10^7^ is obtained at *t* = 0.6, while *min*(*var*(*V*(*t*))) = 0 is obtained at *t* = 20. Hence, it can be said that Fig 6 suggests that the results for the random effects with symmetrical triangular distribution have a smaller variance for *T*(*t*), *I*(*t*) and *V*(*t*).

#### 0.4.3 Confidence intervals

**Fig 7:**
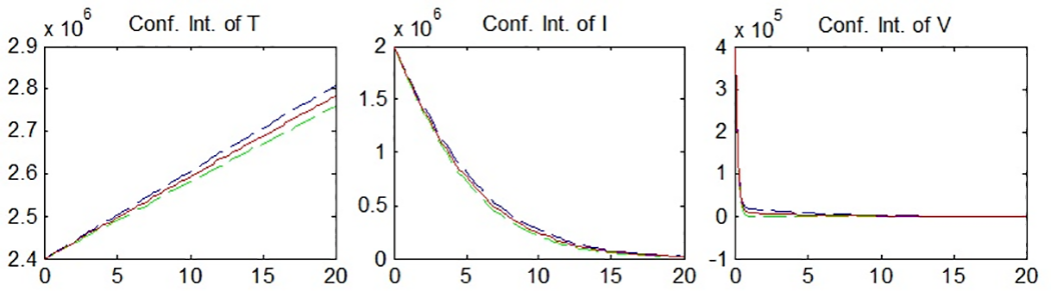
Confidence intervals of *T*(*t*), *I*(*t*) and *V*(*t*).

The confidence intervals of *T*(*t*), *I*(*t*) and *V*(*t*) can be seen in Fig 7 (The dashed lines are the upper and the lower limits of the intervals).

Fig 7 shows narrower confidence intervals for *T*(*t*), *I*(*t*) and *V*(*t*), as a result of the smaller variances. The extremum values of the confidence intervals are (2806800, 20) and (2400000, 0) for *T*(*t*), (2000000, 0) and (13933, 20) for *I*(*t*) and (400000, 0) and (0, 20) for *V*(*t*) (The lower limit of the confidence intervals are calculated by subtracting 3 standard deviations from the means, thus any negative value obtained must be ignored since it is biologically irrelevant).

### 0.5 Comparison of results

More than 10^5^ simulations were made for the model under both normally and symmetrically triangularly varying random effects. Results for solution curves, expectations, variances and confidence intervals were given above. However, the results for standard deviation, second moments, third and fourth central moments, skewness and kurtosis were also calculated from the simulations. When the results for the random effect with normal distribution and symmetrical triangular distribution are compared, some differences can be seen. The higher variance in the results for the normally distributed random effects is the first to be noticed (Table 2).

**Table 2:** Maximum values of the references for normal and triangular distributions, respectively.

Maximum Variance of Results for *T*	40.603 × 10^7^	6.7858 × 10^7^
Maximum Variance of Results for *I*	11.049 × 10^8^	1.8583 × 10^8^
Maximum Variance of Results for *V*	76.700 × 10^6^	12.817 × 10^6^

The results show that there is about 6 times more variance in the normal results compared to the symmetrical triangular results, meaning that the random results for the normal results are 6 times more scattered around the mean values compared to the symmetrically triangularly varying results. The cause of this difference is the characteristics of the distributions used for the random effect. Standard normal distribution has a variance of 1 while the variance of symmetrical triangular distribution in the interval (−1, 1) is $\frac{1}{6}$. The difference in variances causes a difference in standard deviations and also a difference in confidence intervals, as expected. The standard deviation of results for the normally varying random effects are about 2.45 (which is the square-root of 6) times bigger than the standard deviation of the results for the symmetrically triangularly varying effects. As a consequence of the bigger standard deviation, the confidence intervals for the normally distributed effects are larger than the confidence intervals for the symmetrically triangularly distributed effects. Again, these differences can be traced back to the characteristics of the distributions. Further investigation of other characteristics such as higher central moments, skewness and kurtosis yields that while there is not much difference in fourth central moments and kurtosis, there is a noticeable variation between the third central moments and kurtosis, which can be interpreted as the consequence of the properties of the distributions used. Finally, it can be said that the symmetrical triangular distribution could be accepted as more favorable for this study since the scattering of the values would mean too much change in the variables of the model, which could affect the stability of the model.

## 1 Conclusion

In this study, the deterministic stability and the random behavior of the model from [7] were investigated. The disease-free equilibrium *E*^0^ and the endemic equilibrium point *E*^1^ were found. In addition to the equilibrium points, the spectral radius of the system *R*_0_ was found. Once the spectral radius was calculated, the local stability of the model was examined. The results show that the disease-free equilibrium point of the model is locally asymptotically stable if *R*_0_ ≤ 1 and unstable if *R*_0_ > 1. It is also shown that the endemic equilibrium *E*^1^ is locally asymptotically stable if *R*_0_ > 1 and unstable otherwise. A Lyapunov function was constructed for the global stability analysis of the disease free equilibrium. The results show that the disease free equilibrium is globally asymptotically stable provided that *R*_0_ < 1. A geometric approach is used for the global stability analysis of the endemic equilibrium. Thus, it is shown that the endemic equilibrium point *E*^1^ is globally asymptotically stable if *R*_0_ > 1. In the last part, the random behavior of the model is examined. Computer simulations are performed for the model with both normally and triangularly varying random effects. Numerical characteristics of the model such as, expectation, variance and confidence intervals are calculated from the simulations and the distributions for the random effect are compared. It is seen that the random behavior of the model is in compliance with the global and local deterministic stability analysis results. The spectral radius calculated with the parameter values obtained from [7] matches the outcomes of the random simulations.

The stability analysis performed in this study can be used for a wide number of models in different research areas both on local and global scales. Various other probability distributions such as bilateral exponential (Laplace) and generalized beta distributions could be used for the random effect. Furthermore, random behavior analysis and comparison with the deterministic results can be improved by using Brownian motion to form stochastic differential equations for the models. A stochastic model formed by using stochastic differential equations with Brownian motion could provide better results for the accuracy of the equation system in modeling the real process. The methods of this study could be used on models for other diseases and provide a better understanding of the disease dynamics hence making way for better treatment.
